# Myocarditis after COVID‐19 mRNA vaccination: A systematic review of case reports and case series

**DOI:** 10.1002/clc.23828

**Published:** 2022-06-02

**Authors:** Dae Yong Park, Seokyung An, Amandeep Kaur, Saurabh Malhotra, Aviral Vij

**Affiliations:** ^1^ Department of Medicine John H. Stroger Jr Hospital of Cook County Chicago Illinois USA; ^2^ Department of Biomedical Science Seoul National University Graduate School Seoul Korea; ^3^ Department of Pathology McGaw Medical Center at Northwestern University Chicago Illinois USA; ^4^ Division of Cardiology, Cook County Health Chicago Illinois USA; ^5^ Division of Cardiology, Rush Medical College Chicago Illinois USA

**Keywords:** COVID, COVID‐19, myocarditis, vaccine, vaccination

## Abstract

**Background:**

The coronavirus disease of 2019 (COVID‐19) is a global pandemic with over 266 million cases and 5 million deaths worldwide. Anti‐COVID‐19 vaccinations have had exceptional success in subduing the incidence, prevalence, and disease severity of COVID‐19, but rare cases of myocarditis have been reported after COVID‐19 vaccinations.

**Hypothesis:**

Myocarditis occurring after COVID‐19 mRNA vaccinations have distinguishable clinical characteristics. They usually have a favorable prognosis.

**Methods:**

We performed a systematic literature search on PUBMED and MEDLINE database from inception to December 5, 2021. Studies were analyzed based on predetermined eligibility criteria.

**Results:**

A total of 57 studies containing 275 cases of COVID‐19 vaccine‐associated myocarditis were catalogued. Mean age was 26.7 years and male to female ratio was 14:1. For 86.9% of patients, myocarditis occurred after the second dose. Average time to onset and length of hospitalization were 3.7 and 3.9 days, respectively. Prognosis was largely benign, but there was a 1.1% reported mortality. Chest pain (95.2%), elevation of troponin (100%), and ST elevation on electrocardiography (68.5%) were common. Nonsteroidal anti‐inflammatory drugs (81.4%) were the most used medication, followed by colchicine (33.1%).

**Conclusions:**

Patients with COVID‐19 vaccine‐associated myocarditis are usually younger males presenting with chest pain 3–4 days after receiving their second dose of COVID vaccine. Diagnosis is made by exclusion of all other etiologies. Given significant population benefit from COVID‐19 vaccination, physicians should continue to encourage vaccination while remaining vigilant of the very rare occurrence of myocarditis following COVID‐19 vaccination.

**Abbreviations**
BNP: brain natriuretic peptide
CDC: Centers for Disease Control and Prevention
COVID‐19: coronavirus disease of 2019
CRP: C‐reactive protein
ESR: erythrocyte sedimentation rate
LVEF: left ventricular ejection fracture
mRNA: messenger ribonucleic acid
NSAIDs: nonsteroidal anti‐inflammatory drugs
WBC: white blood cell
VAERS: Vaccine Adverse Event Reporting System

## 1 INTRODUCTION

Large multinational efforts have been made to battle the highly contagious SARS‐CoV‐2 virus. Within a year after the COVID‐19 pandemic began, multiple vaccines had been developed, mass produced, and commercialized. On December 11, 2020, the US Food and Drug Administration sanctioned the first emergency use authorization for Pfizer‐BioNTech's COVID‐19 vaccine (BNT162b2), followed by Moderna's COVID‐19 Vaccine (mRNA‐1273) on December 18, 2020, and Janssen's COVID‐19 Vaccine (Ad26. COV2.S) on February 27, 2021.^1^ Since their approval, the vaccines have been administered to millions of people around the world, playing a major role in decreasing the transmissibility and mortality from COVID‐19 among the vaccinated.^2^

While the vaccines have a remarkable safety record, there have been some reports of adverse reactions, ranging from fever, fatigue, and headache to anaphylaxis, thrombosis with thrombocytopenia syndrome, and Guillain–Barre syndrome.^3^ In particular, myocarditis is being recognized as a rare complication of COVID‐19 mRNA vaccinations, a finding also addressed by the Centers for Disease Control and Prevention (CDC).^3^ The CDC had reported a total of 296 million messenger ribonucleic acid (mRNA) COVID‐19 vaccine doses administered by the June of 2021, when the Vaccine Adverse Event Reporting System (VAERS) indicated a total of 1226 reports of myocarditis after mRNA vaccination.^4^ At a similar time, the CDC reported the number of myocarditis cases observed within 7 days after the second dose of the vaccine was significantly higher than expected.^5^, ^6^ Similarly, in May 2021, Vigibase, an international pharmacovigilance database, indicated the occurrence of 1251 vaccine‐associated myocarditis, of which 214 (17.1%) were linked to COVID‐19 vaccines.^7^ With expansion of COVID‐19 vaccination indications to children and need for additional booster vaccinations in adults, we performed an updated systematic review of COVID‐19 vaccine‐associated myocarditis, delineating its demographics and clinical characteristics.

## 2 METHODS

**Figure 1:**
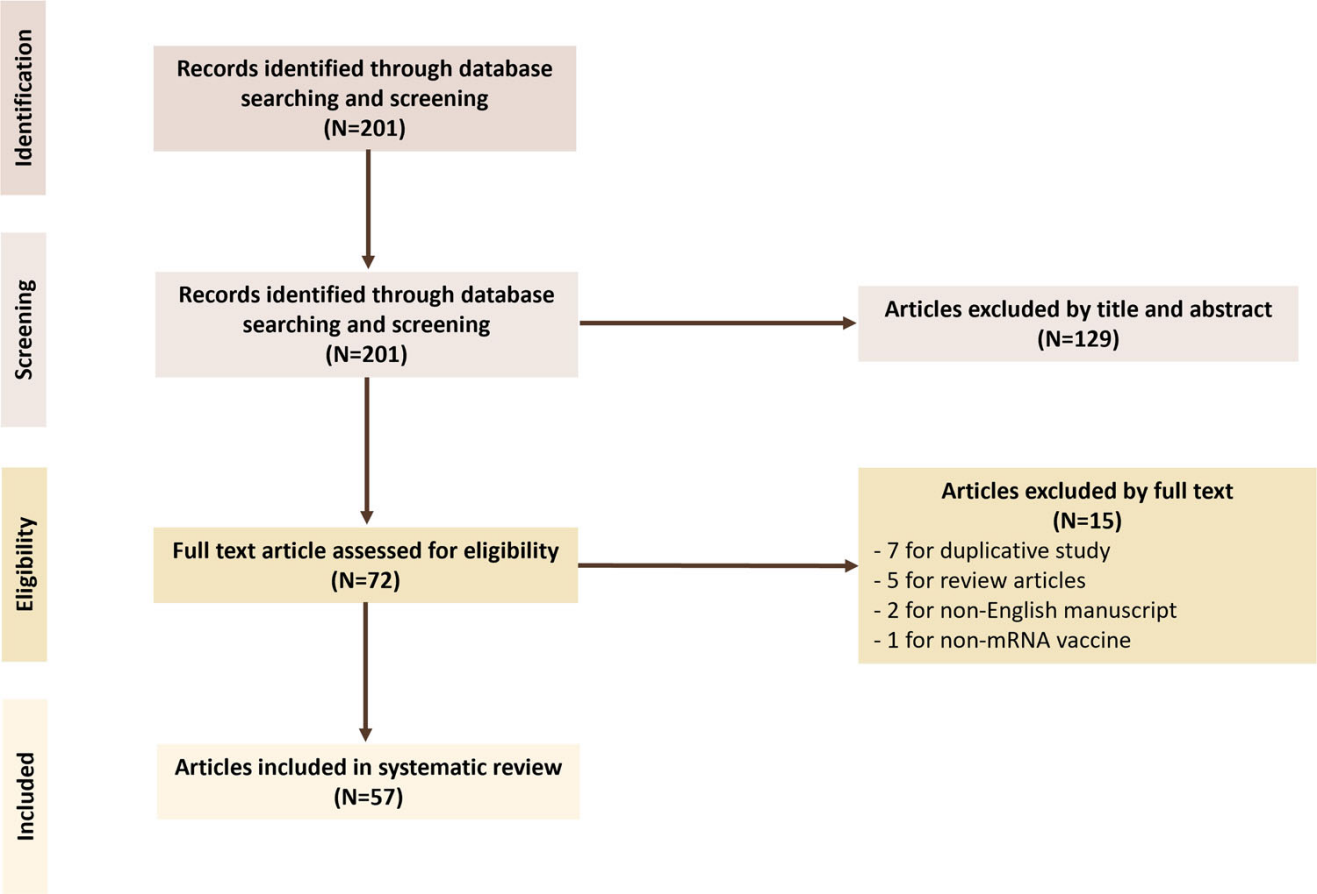
Flow diagram illustrating the systematic review selection process

We conducted a systematic review of the literature on myocarditis occurring after COVID‐19 vaccination using the following search engines: PUBMED and MEDLINE. The search string was as follows: (1) for the outcome of interest, the following keyword was used: “myocarditis” and (2) for exposure, the following keywords were considered: “COVID,” “COVID‐19,” “COVID19,” “SARS‐CoV‐2,” “mRNA,” “vaccine,” and “vaccination.” Cases pertaining to non‐mRNA vaccinations were excluded. We selected all studies in English published up to December 5, 2021, which were individually assessed for eligibility. Duplicative studies on the same population and non‐case reports were excluded. All cases were recorded, studied, and characterized. The PRISMA flow diagram for this study is shown in Figure 1. Included studies are summarized in Table 1 and Table S1.

**Table 1:** Summary of included studies

Author	Year	Country	Cases	Age(s)	Male (%)	Vaccines	Dose^a^	LOS^b^	Outcome
Bautista et al.	2020	Spain	1	39	100	1 BNT162b2	2nd	6	Recovered
Dickey et al.	2021	USA	6	16–40	100	5 BNT162b2, 1 mRNA‐1273	2nd	‐	Recovered
Mansour et al.	2021	USA	2	21–25	50	2 mRNA‐1273	2nd	3 (1), −(1)	Recovered
Nevet et al.	2021	Israel	3	20–24	100	3 BNT162b2	2nd	‐	Recovered
Habib et al.	2021	Qatar	1	37	100%	1 BNT162b2	2nd	6	Recovered
Singh et al.	2021	USA	1	24	100	1 BNT162b2	2nd	4	Recovered
Muthukumar et al.	2021	USA	1	52	100	1 mRNA‐1273	2nd	4	Recovered
Watkins et al.	2021	USA	1	20	100	1 BNT162b2	2nd	‐	Recovered
D'Angelo et al.	2021	Italy	1	30	100	1 BNT162b2	2nd	‐	Recovered
Abu Mouch et al.	2021	Israel	6	16–45	100	6 BNT162b2	2nd (5), 1st (1)	6	Recovered
Albert et al.	2021	USA	1	24	100	1 mRNA‐1273	2nd	‐	Recovered
Montgomery et al.	2021	USA	23	20–51	100	7 BNT162b2, 16 mRNA‐1273	2nd (20), 1st (3)	7 (16), −(7)	Recovered
Rosner et al.	2021	USA	6	19–39	100	5 BNT162b2, 1 mRNA‐1273	2nd (5), 1st (1)	3	Recovered
McLean et al.	2021	USA	1	16	100	1 BNT162b2	2nd	6	Recovered
Kim et al.	2021	USA	4	23–70	75	2 BNT162b2, 2 mRNA‐1273	2nd	‐	Recovered
Shaw et al.	2021	USA	4	16–31	50	3 BNT162b2, 1 mRNA‐1273	2nd (2), 1st (2)	‐	Recovered
Minocha et al.	2021	USA	1	17	100	1 BNT162b2	2nd	6	Recovered
Park et al.	2021	USA	2	15–16	100	2 BNT162b2	2nd (1), 1st (1)	4	Recovered
Marshall et al.	2021	USA	7	14–19	100	7 BNT162b2	2nd	3.7	Recovered
Kim et al.	2021	Korea	1	29	100	1 BNT162b2	2nd	1	Recovered
Gautam et al.	2021	USA	1	66	100	1 BNT162b2	2nd	‐	Recovered
Nguyen et al.	2021	Germany	1	20	100	1 mRNA‐1273	1st	‐	Recovered
Schmitt et al.	2021	France	1	19	100	1 BNT162b2	2nd	1	Recovered
Patrignani et al.	2021	Italy	1	56	100	1 BNT162b2	1st	‐	Recovered
Williams et al.	2021	Canada	1	34	100	1 mRNA‐1273	2nd	5	Recovered
Miqdad et al.	2021	Saudi Arabia	1	18	100	1 BNT162b2	2nd	3	Recovered
Ambati et al.	2021	USA	2	16–17	100	2 BNT162b2	2nd	3	Recovered
Dionne et al.	2021	USA	15	12–18	93.3	15 BNT162b2	2nd (14), 1st (1)	2	Recovered
Ehrlich et al.	2021	Germany	1	40	100	1 BNT162b2	1st	4	Recovered
Kim et al.	2021	Korea	1	24	100	1 BNT162b2	2nd	‐	Recovered
Chelala et al.	2021	USA	5	16–19	100	4 BNT162b2, 1 mRNA‐1273	2nd	4.4	Recovered
Patel et al.	2021	USA	5	19–37	100	4 BNT162b2, 1 mRNA‐1273	2nd (4), 1st (1)	1.8	Recovered
Tailor et al.	2021	USA	1	44	100	1 mRNA‐1273	2nd	5	Recovered
Abbate et al.	2021	USA	2	27–34	50	2 BNT162b2	2nd (1), 1st (1)	73 (1), – (1)	Death (1), Recovered (1)
Larson et al.	2021	USA, Italy	8	21–56	100	5 BNT162b2, 3 mRNA‐1273	2nd (7), 1st (1)	‐	Recovered
Verma et al.	2021	USA	2	42–45	50	1 BNT162b2, 1 mRNA‐1273	1st (1), 2nd (1)	7 (1), – (1)	Death (1), Recovered (1)
Koizumi et al.	2021	Japan	2	22–27	100	2 mRNA‐1273	2nd	4 (1), – (1)	Recovered
Witberg et al.	2021	USA	54	‐	94.4	54 BNT162b2	‐	3	Recovered
Hudson et al.	2021	USA	2	22–24	100	2 BNT162b2	2nd	1.5	Recovered
Starekova et al.	2021	USA	5	17–38	80	3 BNT162b2, 2 mRNA‐1273	2nd	‐	Recovered
Murakami et al.	2021	Japan	2	27–38	100	2 BNT162b2	2nd (1), 1st (1)	9	Recovered
Perez et al.	2021	USA	7	22–71	85.7	3 BNT162b2, 4 mRNA‐1273	2nd (6), 1st (1)	2.6	Recovered
Isaak et al.	2021	Germany	1	15	100	1 BNT162b2	2nd	7	Recovered
Kaul et al.	2021	USA	2	21–28	100	1 BNT162b2, 1 mRNA‐1273	2nd	3	Recovered
Shiyovich et al.	2021	Israel	15	17–76	100	‐	2nd (10), 1st (5)	‐	Recovered
Levin et al.	2021	USA	4	20–30	75	1 BNT162b2, 3 mRNA‐1273	2nd	2.5	Recovered
Visclosky	2021	USA	1	15	100	1 BNT162b2	2nd	‐	Recovered
King et al.	2021	USA	4	20–30	75	1 BNT162b2, 3 mRNA‐1273	2nd	2.3 (3), −(1)	Recovered
Onderko et al.	2021	USA	3	25–36	100	2 BNT162b2, 1 mRNA‐1273	2nd	‐	Recovered
Choi et al.	2021	South Korea	1	22	100	1 BNT162b2	1st	‐	Death
Das et al.	2021	USA	25	12–17	88	25 BNT162b2	2nd (22), 1st (3)	2.7	Recovered
Schauer et al.	2021	USA	13	12–17	92.3	13 BNT162b2	2nd	‐	Recovered
Chamling et al.	2021	Germany	2	20–25	100%	2 BNT162b2	2nd (1), 1st (1)	‐	Recovered
Tano et al.	2021	USA	8	15–17	100%	8 BNT162b2	2nd (7), 1st (1)	‐	Recovered
Matta et al.	2021	USA	1	27	100	1 BNT162b2	2nd	1	Recovered
Alania et al.	2021	Spain	1	28	100	1 BNT162b2	2nd	10	Recovered
Kaneta et al.	2021	Japan	1	25	100	1 mRNA‐1273	2nd	‐	Recovered

^a^ Number within the parenthesis signifies the number of patients within the study corresponding to the said description.

^b^ Average length of stay in days.

We obtained baseline demographics such as age, sex, vaccine type (mRNA‐1273, BNT‐162b2), vaccine dose (first and second), time to onset, length of stay, symptoms, and vital signs at presentation, initial laboratory and testing data, and treatment used in the case studies. Cases that did not report any symptoms were classified as “not reported,” and cases that reported some symptoms were assumed to be negative for those that were not mentioned. Continuous variables were presented as mean with standard deviations, and categorical variables were expressed as numbers and percentages. We employed Wilcoxon rank‐sum test to compare the clinical characteristics of myocarditis occurring after mRNA‐1273 and BNT‐162b2 vaccines. All statistical analyses were performed using SAS 9.4 software (SAS Institute).

## 3 RESULTS

We identified 201 studies relevant to COVID‐19 vaccination and myocarditis. Further review identified a total of 57 studies reporting cases, case series, and summated case series of COVID‐19 vaccine‐associated myocarditis.^8^, ^9^, ^10^, ^11^, ^12^, ^13^, ^14^, ^15^, ^16^, ^17^, ^18^, ^19^, ^20^, ^21^, ^22^, ^23^, ^24^, ^25^, ^26^, ^27^, ^28^, ^29^, ^30^, ^31^, ^32^, ^33^, ^34^, ^35^, ^36^, ^37^, ^38^, ^39^, ^40^, ^41^, ^42^, ^43^, ^44^, ^45^, ^46^, ^47^, ^48^, ^49^, ^50^, ^51^, ^52^, ^53^, ^54^, ^55^, ^56^, ^57^, ^59^, ^60^, ^61^, ^62^, ^63^, ^64^, ^65^ A total of 275 cases of COVID‐19 vaccine‐associated myocarditis were reported. All cases were inpatient encounters. In most cases, a diagnosis of myocarditis was reached after considering clinical presentation and testing data, implementing diagnosis of exclusion, and applying the 2018 Lake‐Louise consensus criteria on imaging findings. A few larger studies utilized CDC's case definition for myocarditis.^66^ Four studies contained histopathologic evidence for myocarditis.^16^, ^21^, ^45^, ^60^

The average age of the patients with myocarditis was 26.7 years, with the youngest patient at 12 years and the oldest at 71 years. Males comprised 93.5% of the patient population, and the male to female ratio was 14:1. More than half (76.0%) of the cases were reported after Pfizer's BNT‐162b2 vaccine and 18.6% after Moderna's mRNA‐1273 vaccine. Excluding the 54 patients whose vaccine dose was not reported, myocarditis occurred after the second dose in 86.9% and after the first dose in 13.1% of the patients. Patients presented after an average of 3.7 days from receiving their last vaccination dose. The earliest time to presentation was 6 h while the longest was 90 days. Patients were hospitalized for an average of 3.9 days. Death occurred in 3 (1.1%) patients (Table 2).

**Table 2:** Clinical characteristics of patients

Characteristics	*N* (%)
Age, mean (range), year	26.7 (12–71)
Sex	
Male	257 (93.5)
Female	18 (6.5)
Vaccine type (*n* = 260)	
BNT‐162b2	209 (80.4)
mRNA‐1273	51 (19.6)
Vaccine dose (*n* = 221)	
First	29 (13.1)
Second	192 (86.9)
Time to onset, mean (SD), day	3.7 (6.31)
Length of stay, mean (SD), day	3.9 (5.53)
Mortality	3 (1.1%)

Excluding the 25 patients whose symptoms were not reported, chest pain (95.2%) was the most common symptom at presentation, followed by fever (30.8%), myalgia (18.0%), and dyspnea (13.2%). Presence of other symptoms are shown in Table 3. The mean body temperature of the patients was 37.4°C; mean systolic blood pressure, 121.9 mmHg; mean diastolic blood pressure, 72.3 mmHg; mean pulse rate, 84.6 beats per minute; mean respiratory rate, 18.2 breaths per minute; and the mean oxygen saturation, 98.0% on room air (Table 3).

**Table 3:** Clinical presentation of patients with myocarditis following COVID‐19 vaccination

Characteristics	*N* (%)	Characteristics	*N* (%)
Symptoms (*n* = 250)		Laboratory and testing variables	
Fever		Troponin (*n* = 170)	
Yes	77 (30.8)	Elevated	170 (100)
No	173 (69.2)	Not elevated	0 (0)
Chest pain		cTnI, mean (SD), ng/ml	9.1 (11.4)
Yes	238 (95.2)	Peak cTnI, mean (SD), ng/ml	11.3 (11.5)
No	12 (4.8)	hs‐cTnI, mean (SD), ng/L	1737 (2516)
Myalgia		Peak hs‐cTnI, mean (SD), ng/L	4759 (5557)
Yes	45 (18.0)	cTnT, mean (SD), ng/L	302 (436)
No	205 (82.0)	Peak cTnT, mean (SD), ng/L	681 (441)
Headache		WBC (*n* = 43)	
Yes	30 (12.0)	Normal	34 (79.1)
No	220 (88.0)	Abnormal	9 (20.9)
Dyspnea		WBC count, mean (SD), /μl	9124 (2886)
Yes	33 (13.2)	CRP (*n* = 147)	
No	217 (86.8)	Elevated	36 (24.5)
Dizziness		Not elevated	111 (75.5)
Yes	2 (0.8)	CRP, mean (SD), mg/L	39 (39)
No	248 (99.2)	ESR (*n* = 42)	
Malaise		Elevated	18 (42.9)
Yes	27 (10.8)	Not elevated	24 (57.1)
No	223 (89.2)	ESR, mean (SD), mm/h	19 (12)
Nausea		BNP (*n* = 34)	
Yes	11 (4.4)	Elevated	8 (23.5)
No	239 (95.6)	Not elevated	26 (76.5)
Vomiting		BNP, mean (SD), pg/ml	86 (137)
Yes	12 (4.8)	Pro‐BNP (*n* = 19)	
No	238 (95.2)	Elevated	11 (57.9)
Diaphoresis		Not elevated	8 (42.1)
Yes	7 (2.8)	BNP, mean (SD), pg/ml	758 (1158)
No	243 (97.2)	ECG (*n* = 232)	
		Normal	42 (18.1)
Vital signs, mean (SD)		ST elevation	159 (68.5)
Temperature, °C	37.4 (0.53)	ST depression	8 (3.4)
SBP, mmHg	121.9 (12.22)	PR depression	21 (9.1)
DBP, mmHg	72.3 (6.71)	T wave inversion	30 (12.9)
PR, beats per minute	84.6 (12.46)	Others^a^	14 (6.0)
RR, breaths per minute	18.2 (3.19)	LVEF (*n* = 167)	
SpO_2_, %	98.0 (1.88)	<50%	28 (16.8)
		≥50%	139 (83.2)
		LVEF, mean (SD), %	54.2 (9.6)

Abbreviations: BNP, brain natriuretic peptide; CRP, C‐reactive protein; cTnI, cardiac troponin I; cTnT, cardiac troponin T; DBP, diastolic blood pressure; ECG, electrocardiogram; ESR, erythrocyte sedimentation rate; hs‐cTnI, highly sensitive cardiac troponin I; LVEF, left ventricular ejection fraction; PR, pulse rate; RR, respiratory rate; SBP, systolic blood pressure; SpO2, pulse oximeter oxygen saturation; WBC, white blood cell.

^a^ Ectopic atrial rhythm, sinus tachycardia, nonsustained ventricular tachycardia, left axis deviation, incomplete right bundle branch block, J‐point elevation, widened QRS, peaked T waves, AV dissociation, sinus bradycardia.

Troponin was elevated in all 170 patients where troponin level was reported. The mean troponin I, high‐sensitivity troponin I, and troponin T levels were 9.1 ng/ml, 1737 pg/ml, and 301.9 ng/L, respectively. Mean WBC, C‐reactive protein (CRP), erythrocyte sedimentation rate (ESR), brain natriuretic peptide (BNP), and pro‐BNP values are summarized in Table 2. Most common electrocardiogram finding was ST elevation (57.8%), followed by normal sinus rhythm (15.3%) and T wave inversion (10.9%). Mean left ventricular ejection fraction was 54.2%.

Among the 118 patients whose information on treatment was available, most common treatment included nonsteroidal anti‐inflammatory drugs (NSAIDs) (81.4%), followed by colchicine (33.1%), beta‐blocker (16.9%), and steroids (15.3%). Nine patients (7.6%) recovered despite receiving no treatment (Table 4).

**Table 4:** Clinical course and treatment of patients with myocarditis following COVID‐19 vaccination

Treatment (*n* = 118)	*N* (%)
NSAID	96 (81.4)
Colchicine	39 (33.1)
Steroids	18 (15.3)
Beta‐blocker	20 (16.9)
IVIG	14 (11.9)
Aspirin	13 (11.0)
ACEi/ARB	14 (11.9)
Acetaminophen	10 (8.5)
Diuretics	4 (3.4)
Spironolactone	4 (3.4)
Vasopressors	3 (2.5)
Anakinra	2 (1.7)
No treatment given	9 (7.6)

Abbreviations: ACEi, angiotensin‐converting enzyme inhibitor; ARB, angiotensin II receptor blocker; IVIG, intravenous immunoglobulin; NSAID, nonsteroidal anti‐inflammatory drug.

The clinical characteristics of myocarditis occurring after Pfizer's BNT162b2 vaccine and after Moderna's mRNA‐1273 vaccine were compared. The average time to onset after the BNT162b2 vaccine was 3.7 ± 7.21 days while that after the mRNA‐1273 vaccine was 3.5 ± 3.84 days (*p* = .14). The average time of hospital stay for BNT162b2 vaccine‐associated myocarditis was 3.6 ± 6.04 days, while that for mRNA‐1273‐associated myocarditis was 4.9 ± 2.20 days (*p* < .01). Troponin I was significantly greater with mRNA‐1273 than BNT162b2 (*p* = .045). WBC, CRP, ESR, and BNP were not statistically different between the two types of mRNA vaccines (Table S2).

## 4 DISCUSSION

Vaccines against SARS‐CoV2 have been developed at an expedited pace and is a testament to the decades of knowledge gained from vaccine development and advances in biomedical technology. Vaccines, and not just the ones against SARS‐CoV2, are associated with rare adverse effects, one of them being myocarditis.^67^ Myocarditis occurring after vaccination is not an unprecedented phenomenon as demonstrated by the association of smallpox vaccine with myocarditis.^68^ One retrospective study in Israel postulated the incidence of myocarditis as 1 every 26 000 males and 1 every 218 000 females after the second COVID vaccine, having a rate ratio of 2.35 compared with unvaccinated persons.^69^ Another study of VAERS from December 2020 to August 2021 reported the incidence rate of 5.98 cases of myocarditis per million COVID vaccine doses.^70^

The molecular makeup of prior vector‐based vaccines and the current mRNA‐lipid nanoparticles‐based vaccines (BNT162b2 and mRNA‐1273) are different.^71^ In the past, immune‐mediated mechanisms were implicated for myocarditis due to vaccines. Similarly, antibody‐mediated hypersensitivity and delayed‐type hypersensitivity may be responsible for myocarditis after mRNA vaccines and could explain the occurrence of myocarditis after predominantly (86.9%) second dose of COVID‐19 vaccination in most patients.^72^ The second dose of vaccines was typically scheduled 21–28 days after the first dose, allowing sufficient time for delayed‐type hypersensitivity to develop. Given the presence of prior exposure and the interval time, either the first dose of the vaccine or in some cases previous COVID‐19 infection may have exposed an epitope, priming the immune system against an entity in the myocardium via molecular mimicry or cross‐reaction.^73^

An alternate mechanism by which mRNA vaccines can elicit immunological response is by directly binding to pattern recognition receptors intracellularly.^73^ For example, Toll‐like receptors and retinoic acid‐inducible gene‐I are known to recognize RNA in endosomes and cytosol, respectively, leading to cascades that activate cytokines and translate pro‐inflammatory agents.^73^ A recent study performed endometrial biopsy in two patients, consistently revealing inflammatory infiltrate consisting of macrophages, T cells, eosinophils, and B cells.^60^ Another study on biopsy‐proven lymphocytic myocarditis following mRNA COVID‐19 vaccination reported myocytes necrosis, CD68‐positive macrophages, and numerous CD3‐positive T cells, further corroborating the hypothesis that post‐vaccine myocarditis occurs via an immunologic response.^21^

The mean age of the 275 patients with myocarditis post‐COVID‐19 vaccination was 26.7 years, 93.5% of them were male. This predisposition to younger population may be due to stronger immune responses or immune naivety in the young.^35^ Previous studies have reported myocarditis to occur at a greater frequency in males, with a male to female ratio around 1.5:1 to 1.7:1^74^, ^75^, ^76^ This differences in gender were thought to be due to genetics, effects of testosterone on the heart, influence of the sex hormones on the immune system, and difference in immune responses.^77^

Comparison of the clinical course of myocarditis occurring after Pfizer's BNT162b2 vaccine and that after Moderna's mRNA‐1273 vaccine suggested longer length of hospital stay and greater troponin I level in the latter. Although definite conclusions cannot be made on the differences in the severity of myocarditis between the two mRNA‐based vaccines, the finding is consistent with that of a Danish study, which noted a significantly increased risk of myocarditis or myopericarditis after mRNA‐1273 as opposed to a nonsignificantly increased risk after BNT162b2^65^ The rate of myocarditis or myopericarditis was also more than threefold higher after mRNA‐1273 compared to BNT162b2.^65^ These findings suggest that myocarditis occurring after mRNA‐1273 may be more severe than that after BNT162b2, but the underlying mechanism remains elusive.

While the authors of the included case reports have hinted at a strong association between COVID‐19 vaccines and myocarditis, a definite causal relationship cannot be established based on available data. Phase 3 trials of COVID‐19 mRNA vaccines did not detect any major vaccine‐associated myocarditis.^78^, ^79^ While there maybe apprehension among some patients and providers, the benefits of COVID‐19 vaccination at preventing severe COVID‐19 infections cannot be emphasized enough. Advisory Committee on Immunization Practices (ACIP) performed an individual‐level assessment which concluded that the benefits of vaccination clearly outweighed the risks by preventing more than 11 000 COVID‐19 cases, 560 hospitalizations, 138 intensive care unit admissions, and 6 deaths per million doses of vaccines compared to 39–47 expected myocarditis cases after vaccination in males aged 12–29 years.^4^ Vaccine recipients, providers, and healthcare workers should be aware of this very rare adverse effect of myocarditis after COVID‐19 vaccination, but this should not be a reason to deter vaccination.

There are many unanswered questions. The true incidence of myocarditis after COVID‐19 vaccinations is still unclear as association does not equal causation. Additionally, larger scale studies with longer follow ups are needed to assess long‐term risks of cardiomyopathies or arrhythmias in those with vaccine‐associated myocarditis? Duration of follow‐up, safety of subsequent mRNA vaccine booster doses need to be addressed. Perhaps closer monitoring for 1 month should be practiced in patients with a history of COVID‐vaccine‐related myocarditis given that more than 99% of the cases occurred within 30 days from vaccination.^54^

This systematic review inherently has many limitations. The total number of cases is relatively small as myocarditis after vaccination is a rare adverse event, and it has been only about a year since vaccines have rolled out. There is a possibility of selection bias where milder forms of COVID‐19 vaccine‐associated myocarditis may not have been reported or identified due to milder and nonspecific symptoms. There is also the possibility of older individuals with myocarditis being diagnosed with ischemic heart diseases as they tend to have more cardiovascular risk factors. Only published data including inpatient cases were included in the study. Clinical evaluations varied, and some authors omitted subjective symptoms and many of the objective values. The clinical workup was especially heterogeneous. Nonstandardized parameters and omissions imposed a great challenge in comparing the cases.

## 5 CONCLUSION

Myocarditis is an extremely rare adverse effect that has been reported after COVID‐19 vaccination; however, it should be considered as a differential in young adults with chest pain, fever, and dyspnea 3–4 days after vaccination. While patients should be informed of this rare occurrence, they should continue to receive counselling and education regarding the significant individual and population level benefits of COVID‐19 vaccination.

## AUTHOR CONTRIBUTIONS

**Dae Yong Park** and **Seokyung An**: Conceptualization, data curation, formal analysis, investigation, methodology, software, visualization, writing original draft, review, and editing. **Amandeep Kaur**: Review and editing. **Saurabh Malhotra**: Supervision, validation, review and editing. **Aviral Vij**: Project administration, resources, supervision, validation, review and editing.

## CONFLICTS OF INTEREST

The authors declare no conflicts of interest.

## Supporting information

Supplementary information.

## Data Availability

Data included in this study are publicly available as published articles in the literature.
