# Supplementary material for: Myocarditis after COVID‐19 mRNA vaccination: A systematic review of case reports and case series
Source: Clin Cardiol. 2022 Jun 2;45(7):691–700. doi: 10.1002/clc.23828 (PMC9286338; doi:10.1002/clc.23828)
Supplement: Supplementary file 1 — Supplementary information. [file CLC-45-691-s001.docx]

Supplementary Table 1. Summary of included studies

| **Author** | **Year** | **County** | **Cases** | **Age(s)** | **Male** | **Vaccines** | **Dose** | **Symptoms** | **Treatment** | **LOS*** | **Outcome** |
| --- | --- | --- | --- | --- | --- | --- | --- | --- | --- | --- | --- |
| Bautista et al. | 2020 | Spain | 1 | 39 | 100% | 1 BNT162b2 | 2^nd^ | Fever, chest pain | - | 6 | Recovered |
| Dickey et al. | 2021 | USA | 6 | 16-40 | 100% | 5 BNT162b2,  1 mRNA-1273 | 2^nd^ | Fever (2), chest pain (6),  myalgia (4), headache (2),  malaise (1) | - | - | Recovered |
| Mansour et al. | 2021 | USA | 2 | 21-25 | 50% | 2 mRNA-1273 | 2^nd^ | Fever (1), chest pain (2),  dizziness (1) | Metoprolol (1)** | 3 (1)  - (1) | Recovered |
| Nevet et al. | 2021 | Israel | 3 | 20-24 | 100% | 3 BNT162b2 | 2^nd^ | Fever, chest pain | Ibuprofen, colchicine | - | Recovered |
| Habib et al. | 2021 | Qatar | 1 | 37 | 100% | 1 BNT162b2 | 2^nd^ | Chest pain, headache, myalgia | Acetaminophen | 6 | Recovered |
| Singh et al. | 2021 | USA | 1 | 24 | 100% | 1 BNT162b2 | 2^nd^ | Fever, chest pain, headache | - | 4 | Recovered |
| Muthukumar et al. | 2021 | USA | 1 | 52 | 100% | 1 mRNA-1273 | 2^nd^ | Chest pain, headache, myalgia | Carvedilol, lisinopril | 4 | Recovered |
| Watkins et al. | 2021 | USA | 1 | 20 | 100% | 1 BNT162b2 | 2^nd^ | Chest pain, dyspnea | Ibuprofen, colchicine, metoprolol | - | Recovered |
| D’Angelo et al. | 2021 | Italy | 1 | 30 | 100% | 1 BNT162b2 | 2^nd^ | Fever, chest pain, nausea, diaphoresis | Prednisolone, bisoprolol, aspirin | - | Recovered |
| Abu Mouch et al. | 2021 | Israel | 6 | 16-45 | 100% | 6 BNT162b2 | 2^nd^ (5)  1^st^ (1) | - | NSAID, colchicine | 6 | Recovered |
| Albert et al. | 2021 | USA | 1 | 24 | 100% | 1 mRNA-1273 | 2^nd^ | Fever, chest pain, myalgia | - | - | Recovered |
| Montgomery et al. | 2021 | USA | 23 | 20-51 | 100% | 7 BNT162b2,  16 mRNA-1273 | 2^nd^ (20)  1^st^ (3) | Chest pain | - | 7 (16)  - (7) | Recovered |
| Rosner et al. | 2021 | USA | 6 | 19-39 | 100% | 5 BNT162b2,  1 mRNA-1273 | 2^nd^ (5)  1^st^ (1) | Fever (2), chest pain (6) | Ibuprofen (3), colchicine (3), steroids (1), BB (2), ARB (1) | 3 | Recovered |
| McLean et al. | 2021 | USA | 1 | 16 | 100% | 1 BNT162b2 | 2^nd^ | Fever, chest pain, myalgia | Ibuprofen, IVIG | 6 | Recovered |
| Kim et al. | 2021 | USA | 4 | 23-70 | 75% | 2 BNT162b2,  2 mRNA-1273 | 2^nd^ | Fever (3), chest pain (4), myalgia (3), dyspnea (3), diaphoresis (1) | NSAID (1), steroid (1), colchicine (2), none (1) | - | Recovered |
| Shaw et al. | 2021 | USA | 4 | 16-31 | 50% | 3 BNT162b2,  1 mRNA-1273 | 2^nd^ (2)  1^st^ (2) | Chest pain (1) | - | - | Recovered |
| Minocha et al. | 2021 | USA | 1 | 17 | 100% | 1 BNT162b2 | 2^nd^ | Fever, chest pain, myalgia | NSAID | 6 | Recovered |
| Park et al. | 2021 | USA | 2 | 15-16 | 100% | 2 BNT162b2 | 2^nd^ (1)  1^st^ (1) | Fever (1), chest pain (2), headache (1) | IVIG (1), none (1) | 4 | Recovered |
| Marshall et al. | 2021 | USA | 7 | 14-19 | 100% | 7 BNT162b2 | 2^nd^ | Fever (2), chest pain (7), myalgia (1), headache (1), dyspnea (3), nausea (3), vomit (2), malaise (2) | NSAID (6), prednisone (4), colchicine (1), IVIG (3), aspirin (2), furosemide (1) | 3.7 | Recovered |
| Kim et al. | 2021 | Korea | 1 | 29 | 100% | 1 BNT162b2 | 2^nd^ | Chest pain | NSAID, prednisolone | 1 | Recovered |
| Gautam et al. | 2021 | USA | 1 | 66 | 100% | 1 BNT162b2 | 2^nd^ | Chest pain, diaphoresis, vomit | - | - | Recovered |
| Nguyen et al. | 2021 | Germany | 1 | 20 | 100% | 1 mRNA-1273 | 1^st^ | Fever, chest pain, myalgia, malaise | - | - | Recovered |
| Schmitt et al. | 2021 | France | 1 | 19 | 100% | 1 BNT162b2 | 2^nd^ | Chest pain, headache, malaise | None | 1 | Recovered |
| Patrignani et al. | 2021 | Italy | 1 | 56 | 100% | 1 BNT162b2 | 1^st^ | Epigastric pain, diaphoresis, hypotension | - | - | Recovered |
| Williams et al. | 2021 | Canada | 1 | 34 | 100% | 1 mRNA-1273 | 2^nd^ | Fever, chest pain, myalgia | Colchicine, bisoprolol, ramipril, aspirin | 5 | Recovered |
| Miqdad et al. | 2021 | Saudi Arabia | 1 | 18 | 100% | 1 BNT162b2 | 2^nd^ | Chest pain | Colchicine, ramipril, aspirin | 3 | Recovered |
| Ambati et al. | 2021 | USA | 2 | 16-17 | 100% | 2 BNT162b2 | 2^nd^ | Fever (1), myalgia (1), headache (1), malaise (1) | Ibuprofen | 3 | Recovered |
| Dionne et al. | 2021 | USA | 15 | 12-18 | 93.3% | 15 BNT162b2 | 2^nd^ (14)  1^st^ (1) | Fever (10), chest pain (15), myalgia (8), headache (6), fatigue (6) | - | 2 | Recovered |
| Ehrlich et al. | 2021 | Germany | 1 | 40 | 100% | 1 BNT162b2 | 1^st^ | Fatigue, chest pain, headache, dyspnea | BB, ACE-I, aspirin, spironolactone | 4 | Recovered |
| Kim et al. | 2021 | Korea | 1 | 24 | 100% | 1 BNT162b2 | 2^nd^ | Chest pain, myalgia, malaise | - | - | Recovered |
| Chelala et al. | 2021 | USA | 5 | 16-19 | 100% | 4 BNT162b2  1 mRNA-1273 | 2^nd^ | - | NSAID (1), colchicine (1), metoprolol (2), aspirin (1), none (1) | 4.4 | Recovered |
| Patel et al. | 2021 | USA | 5 | 19-37 | 100% | 4 BNT162b2  1 mRNA-1273 | 2^nd^ (4)  1^st^ (1) | Fever (1), chest pain (5), myalgia (3), headache (4), dyspnea (4), malaise (3), nausea (2), vomit (1), diaphoresis (1) | Ibuprofen (1), colchicine (4), lisinopril (1), metoprolol (1), aspirin (1), none (1) | 1.8 | Recovered |
| Tailor et al. | 2021 | USA | 1 | 44 | 100% | 1 mRNA-1273 | 2^nd^ | Chest pain, myalgia, headache, dyspnea, malaise | Colchicine, BB, ACE-I, diuretic | 5 | Recovered |
| Abbate et al. | 2021 | USA | 2 | 27-34 | 50% | 2 BNT162b2 | 2^nd^ (1)  1^st^ (1) | Nausea, vomit | Methylprednisolone, IVIG, vasopressors, anakinra, ventilator, RRT | 73 (1)  - (1) | Death (1)  Recovered (1) |
| Larson et al. | 2021 | USA (4)  Italy (4) | 8 | 21-56 | 100% | 5 BNT162b2  3 mRNA-1273 | 2^nd^ (7)  1^st^ (1) | Fever (8), chest pain (8), myalgia (2), dyspnea (1), cough (1) | NSAID (3), prednisone (2), colchicine (2), none (3) | - | Recovered |
| Verma et al. | 2021 | USA | 2 | 42-45 | 50% | 1 BNT162b2  1 mRNA-1273 | 1^st^ (1)  2^nd^ (1) | Fever (1), chest pain (1), dyspnea (2), dizziness (1) | Methylprednisolone, metoprolol, lisinopril, diuretic, spironolactone | 7 (1)  - (1) | Death (1)  Recovered (1) |
| Koizumi et al. | 2021 | Japan | 2 | 22-27 | 100% | 2 mRNA-1273 | 2^nd^ | Chest pain | Ibuprofen | 4 (1)  - (1) | Recovered |
| Witberg et al. | 2021 | USA | 54 | - | 94.4% | 54 BNT162b2 | - | Fever (5), chest pain (44), dyspnea (3) | - | 3 | Recovered |
| Hudson et al. | 2021 | USA | 2 | 22-24 | 100% | 2 BNT162b2 | 2^nd^ | Fever (2), chest pain (2), nausea (1), vomit (2), diaphoresis (1) | Ibuprofen (1), colchicine (2), aspirin (1) | 1.5 | Recovered |
| Starekova et al. | 2021 | USA | 5 | 17-38 | 80% | 3 BNT162b2  2 mRNA-1273 | 2^nd^ | Fever (3), chest pain (5), myalgia (2), headache (2), dyspnea (2), malaise (4), nausea (2) | - | - | Recovered |
| Murakami et al. | 2021 | Japan | 2 | 27-38 | 100% | 2 BNT162b2 | 2^nd^ (1)  1^st^ (1) | Fever, chest pain | Ibuprofen, colchicine | 9 | Recovered |
| Perez et al. | 2021 | USA | 7 | 22-71 | 85.7% | 3 BNT162b2  4 mRNA-1273 | 2^nd^ (6)  1^st^ (1) | Fever (1), chest pain (7), myalgia (2), dyspnea (3), malaise (2), diaphoresis (1) | Ibuprofen (1), prednisone (2), colchicine (4), metoprolol (4), lisinopril (3), aspirin (1) | 2.6 | Recovered |
| Isaak et al. | 2021 | Germany | 1 | 15 | 100% | 1 BNT162b2 | 2^nd^ | Fever, myalgia | - | 7 | Recovered |
| Kaul et al. | 2021 | USA | 2 | 21-28 | 100% | 1 BNT162b2  1 mRNA-1273 | 2^nd^ | Fever (2), chest pain (2), myalgia (1), headache (1) | None | 3 | Recovered |
| Shiyovich et al. | 2021 | Israel | 15 | 17-76 | 100% | - | 2^nd^ (10)  1^st^ (5) | Fever (2), chest pain (13), malaise (2) | - | - | Recovered |
| Levin et al. | 2021 | USA | 4 | 20-30 | 75% | 1 BNT162b2  3 mRNA-1273 | 2^nd^ | Chest pain (4), malaise (1) | - | 2.5 | Recovered |
| Visclosky | 2021 | USA | 1 | 15 | 100% | 1 BNT162b2 | 2^nd^ | Fever, chest pain, headache, dyspnea, | - | - | Recovered |
| King et al. | 2021 | USA | 4 | 20-30 | 75% | 1 BNT162b2  3 mRNA-1273 | 2^nd^ | Chest pain | - | 2.3 (3)  - (1) | Recovered |
| Onderko et al. | 2021 | USA | 3 | 25-36 | 100% | 2 BNT162b2  1 mRNA-1273 | 2^nd^ | Chest pain (4),  myalgia (2), malaise (2) | Ibuprofen (1), colchicine (1), BB (3) | - | Recovered |
| Choi et al. | 2021 | South Korea | 1 | 22 | 100% | 1 BNT162b2 | 1^st^ | Chest pain | - | - | Death |
| Das et al. | 2021 | USA | 25 | 12-17 | 88% | 25 BNT162b2 | 2^nd^ (22)  1^st^ (3) | Fever (5), chest pain (24), dyspnea (3), vomit (3) | Ibuprofen (21),  IVIG (2), enalapril (2),  spironolactone (2) | 2.7 | Recovered |
| Schauer et al. | 2021 | USA | 13 | 12-17 | 92.3% | 13 BNT162b2 | 2^nd^ | Fever (5), chest pain (11), myalgia (6), headache (3), dyspnea (5), malaise (1), vomit (1) | NSAID (13), steroid (2), IVIG (3) | - | Recovered |
| Chamling et al. | 2021 | Germany | 2 | 20-25 | 100% | 2 BNT162b2 | 2^nd^ (1)  1^st^ (1) | Chest pain | - | - | Recovered |
| Tano et al. | 2021 | USA | 8 | 15-17 | 100% | 8 BNT162b2 | 2^nd^ (7)  1^st^ (1) | Fever (1), chest pain (3), headache (1), cough (1) | - | - | Recovered |
| Matta et al. | 2021 | USA | 1 | 27 | 100% | 1 BNT162b2 | 2^nd^ | Chest pain | Aspirin | 1 | Recovered |
| Alania et al. | 2021 | Spain | 1 | 28 | 100% | 1 BNT162b2 | 2^nd^ | Fever, chest pain, myalgia, headache, dyspnea, malaise, diarrhea | Aspirin | 10 | Recovered |
| Kaneta et al. | 2021 | Japan | 1 | 25 | 100% | 1 mRNA-1273 | 2^nd^ | Fever, chest pain | - | - | Recovered |

*Average length of stay in days

**Number within the parenthesis signifies the number of patients within the study corresponding to the said description

Abbreviations: ACE-I = angiotensin-converting enzyme inhibitor; ARB = angiotensin receptor blockers; BB = beta-blocker; LOS = length of stay; NSAID = non-steroidal anti-inflammatory drug; RRT = renal replacement therapy

Supplementary Table 2. Comparison of myocarditis occurring after BNT162b2 and mRNA-1273

|  | **BNT162b2** | **mRNA-1273** | ***p*-value** |
| --- | --- | --- | --- |
|  | Mean ± SD | Mean ± SD |  |
| Age (Years) | 23.1 ± 8.93 | 29.1 ± 11.02 | <.001 |
| Male (%) | 197 (94.3) | 45 (88.2) | 0.129 |
| Time to Onset (Days) | 3.7 ± 7.21 | 3.5 ± 3.84 | 0.139 |
| Length of Stay (Days) | 3.6 ± 6.04 | 4.9 ± 2.20 | <.001 |
| Temperature (℃) | 37.4 ± 0.42 | 37.7 ± 1.17 | 0.224 |
| Troponin I (ng/mL) | 8.5 ± 11.34 | 12.5 ± 11.17 | 0.045 |
| hs-Troponin I (pg/mL) | 1,908.1 ± 2,768.4 | 1,110.3 ± 1,436.5 | 0.378 |
| WBC (/μL) | 8,920.9 ± 3,657.2 | 9,930.0 ± 3,583.3 | 0.444 |
| CRP (mg/L) | 42.0 ± 39.73 | 44.6 ± 38.02 | 0.152 |
| ESR (mm/hr) | 19.0 ± 12.61 | 18.0 ± 8.14 | 0.500 |
| BNP (pg/mL) | 87.1 ± 140.69 | 67.5 ± 41.72 | 0.187 |
| LVEF (%) | 55.1 ± 10.08 | 51.4 ± 10.12 | 0.005 |

Abbreviations: BNP = brain natriuretic peptide; CRP = C-reactive protein; ESR = erythrocyte sedimentation rate; hs = high sensitivity; LVEF = left ventricular ejection fraction; WBC = white blood cell
